# Is Starch Intake Associated With Periodontal Status? An 11‐Year Longitudinal Analysis Among Finnish Adults

**DOI:** 10.1111/jcpe.14072

**Published:** 2024-09-24

**Authors:** F. H. Jangda, A. L. Suominen, A. Lundqvist, S. Männistö, A. Golkari, E. Bernabé

**Affiliations:** ^1^ Institute of Dentistry Queen Mary University of London London UK; ^2^ Institute of Dentistry University of Eastern Finland Kuopio Finland; ^3^ Oral Health Teaching Unit Kuopio University Hospital Kuopio Finland; ^4^ National Institute for Health and Welfare Helsinki Finland

**Keywords:** adult, diet, nutrition assessment, periodontal diseases, starch

## Abstract

**Aim:**

To evaluate the association between baseline starch intake (amount and sources) and changes in periodontal status over 11 years in adults.

**Methods:**

Adults aged 30–82 years, who participated in the Finnish Health 2000 survey and were re‐examined in 2004/2005 and/or 2011 were included in the study. The consumption of total starch and six relevant food groups (potatoes, fried potatoes, roots and tubers, pasta, wholegrains and legumes) over the past year was determined at baseline with a validated food frequency questionnaire. The number of teeth with periodontal pocketing ≥ 4 mm (NTPP) was recorded during clinical examinations in 2000, 2004/2005 and 2011. The association between baseline starch intake and the 11‐year change in the NTPP was tested in mixed‐effects negative binomial regression models, adjusting for covariates.

**Results:**

A total of 1369 adults were included in the analysis. The mean NTPP was 4.1 ± 5.6, 6.3 ± 5.6, and 4.8 ± 5.9 in waves 1, 2 and 3, respectively. Baseline starch intake (in g/day or % energy intake) was not associated with changes in the NTPP after adjustment for covariates. In analysis by food groups, the baseline intake of wholegrains was negatively associated with the NTPP at baseline.

**Conclusion:**

This study found no evidence of an association between baseline starch intake and changes in periodontal status. Baseline intake of wholegrains was associated with better periodontal status at baseline.

## 1 Introduction

Starch serves as the primary carbohydrate in the human diet (Apriyanto, Compart, and Fettke 2022; Cummings and Stephen 2007). It is a significant source of glucose and plays a crucial role in influencing post‐meal variations in blood glucose levels (Jenkins et al. 1981; Slavin 2013). The classification of starch is based on its resistance to human digestive enzymes (H. N. Englyst, Kingman, and Cummings 1992). Rapidly digestible starch (RDS) is swiftly converted into glucose in the small intestine and is present in freshly cooked starchy foods and highly processed starches. Slowly digestible starch (SDS) undergoes a slower digestion process in the small intestine and is commonly found in all varieties of pasta, nuts and seeds. Resistant starch (RS) does not undergo digestion in the small intestine and can be found in wholegrains and legumes (Atkinson et al. 2021; H. N. Englyst, Kingman, and Cummings 1992; K. N. Englyst and Englyst 2005). There are no current quantitative recommendations for starch intake. Instead, it is recommended that carbohydrate intake should primarily come from wholegrains, vegetables, fruits and legumes (Sonestedt and Øverby 2023; WHO 2023).

A systematic review, conducted to inform the World Health Organization's guideline on carbohydrate intake for adults and children (WHO 2023), found no studies on the association between starch intake and periodontal disease (Halvorsrud et al. 2019). However, the review found very low quality evidence (one cohort and one cross‐sectional study) suggesting an association between intake of wholegrain foods and lower risk of periodontitis (Halvorsrud et al. 2019). The cohort study followed 34,160 male U.S. health professionals, aged 40–75 years, updating their medical and lifestyle information (including history of professionally diagnosed periodontal disease with bone loss) every 2 years and their diet every 4 years between 1986 and 1998. The findings showed that greater intakes of wholegrains and refined grains were each associated with lower periodontal risk, after adjusting for age, health behaviours and body mass index. However, only the intake of wholegrains remained associated with periodontal risk after mutual adjustment (Merchant et al. 2006). Two recent cross‐sectional studies, published after the release of the WHO‐commissioned systematic review, found that greater consumption of nuts, but not of wholegrains or refined grains, was associated with better periodontal parameters among U.S. adults aged 20–55 years (DeMayo et al. 2021) and that consuming only multigrain rice was associated with lower odds of having periodontitis compared to consuming only white rice among Korean adults aged 19–64‐years (Ryu et al. 2023).

The present study addresses current gaps in knowledge in this area and limitations in previous analyses. To that end, we will assess both total starch intake and common sources of starch (including wholegrains) in the Finnish diet, analyse three waves of periodontal data over a decade and control for common determinants of diet and periodontitis. The findings can inform future recommendations regarding the consumption of starch. The aim of this study was to evaluate the relationship between starch intake and changes in periodontal pocketing among adults over an 11‐year period.

## 2 Materials and Methods

### 2.1 Study Population

**FIGURE 1:**
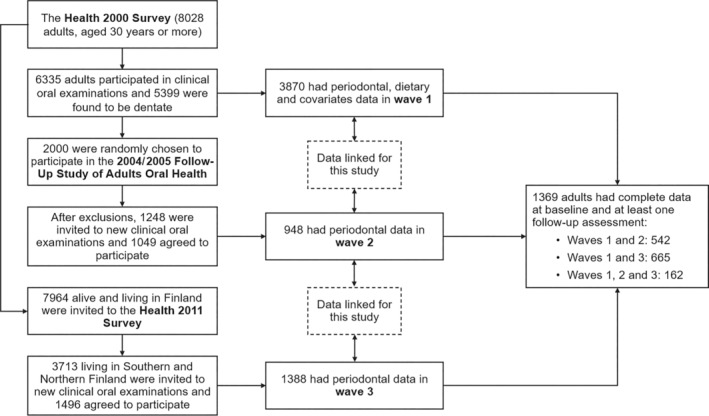
Data linkage for the three surveys of Finnish adults used in this study.

This longitudinal analysis pooled data from individuals who took part in at least two out of three consecutive surveys carried out by the Finnish Institute for Welfare and Health (THL, Figure 1). Baseline data (wave 1) were from the national Health 2000 survey, which recruited 8028 adults aged 30 years or older living in mainland Finland. Overall, 6986 adults (87%) participated in home interviews and 6354 (79%) underwent health examinations. Of those examined, 6335 received dental examinations and 5255 were found to be dentate (Heistaro 2008). Wave 2 data were from the 2004/2005 Follow‐Up study of Adult Oral Health, for which 2000 adults were randomly recruited from those who underwent dental examinations in the Health 2000 survey. People who died or were edentate and those who lived in areas where < 15 participants were sampled for the Health 2000 survey (for logistic reasons) were excluded. After exclusions, 1248 adults were invited for a new dental examination and 1049 agreed to participate (84%). Wave 3 data were from the national Health 2011 survey, for which all participants of the Health 2000 survey alive and living in mainland Finland were invited to participate. Dental examinations were carried out in two of the five recruitment areas (Southern or Northern Finland), with 3281 adults invited and 1524 re‐examined (46%) (Lundqvist, Koponen, and Mäki‐Opas 2011).

Each survey was ethically approved by the Ethics Committee at the Hospital District of Helsinki and Uusimaa. All participants provided written informed consent before participation.

### 2.2 Measures

Periodontal pocketing was the main outcome, which was determined from clinical dental examinations carried out by calibrated dentists following the same protocol in every wave. Participants were seated on a dental chair and clinically examined using a fibre optic light source and WHO periodontal probe with a ball end and markings at 3.5 and 5.5 mm. All teeth were periodontally examined, except for third molars and residual roots. The depth of periodontal pockets was measured at four points (distal angle and midpoint on the buccal side, midpoint on the lingual side and mesial angle) around each tooth, and the deepest measurement was recorded as 0–3, 4–6 or > 6 mm. The *κ*‐values in 2000 for the inter‐ and intra‐examiner reliability, under field conditions, were 0.41 and 0.83 at tooth level, respectively (Suominen‐Taipale et al. 2004, 2008). The number of teeth with periodontal pocket ≥ 4 mm (henceforth referred to as the NTPP for brevity) was estimated for each participant in every wave and treated as a repeated outcome for analysis. In supplemental analysis, we also estimated progression in periodontal pocketing by examining tooth‐by‐tooth transition in codes across waves. A tooth was considered as experiencing progression if there was a move to a higher code or the tooth was lost in subsequent examinations (coded as 1), stable if there was no change in code over time (coded as 0) and experiencing regression if there was a move to a lower code in subsequent examinations (coded as −1). Transition scores for all teeth were summed to calculate the number of teeth with progression in periodontal pocketing.

Starch intake at baseline (wave 1) was the exposure of interest. A validated, semi‐structured food frequency questionnaire (FFQ) was used to measure habitual dietary intake in the past year (Kaartinen et al. 2010; Mannisto et al. 1996; Paalanen et al. 2006). The FFQ comprised 128 commonly used or nutritionally important food items and mixed dishes, which were presented to participants in 12 sections: dairy products; cereals; spreads; vegetables; potatoes, rice and pasta; meat; fish; chicken, turkey, and eggs; fruits and berries; desserts; sweets and snacks; and beverages. A standard portion size was assigned to each FFQ item using natural units (piece, slice, glass, tablespoon, etc.). Each FFQ item had nine response options (never or rarely; 1–3 times per month; once per week; 2–4 times per week; 5–6 times per week; once a day; 2–3 times a day; 4–5 times a day; and more than six times a day). The FFQ was completed at home and sent to THL. When returned, they were checked for unreliable and missing answers by a nutritionist. Responses were used to derive the intake of macronutrients (total starch intake and energy intake [EI]) and food groups using the Finnish Food Composition Database (Fineli, THL). Total starch intake was analysed in quintiles of amount (g/day) and percent of EI (%EI). Based on existing literature (Atkinson et al. 2021; H. N. Englyst, Kingman, and Cummings 1992; K. N. Englyst and Englyst 2005), six common food sources of starch were included in the analysis: (i) potatoes, (ii) potato products (i.e., French fries, potato chips), (iii) roots and tubers, (iv) pasta, (v) legumes and (vi) wholegrains (e.g., barley, oat and rye). All food sources were analysed as quintiles of amount consumed (g/day). Energy adjustment was performed using the standard multivariate method (Willett, Howe, and Kushi 1997), which controls for other dietary sources of energy.

Baseline data on sociodemographic characteristics (sex, age, education and marital status), health behaviours (smoking, alcohol consumption, physical activity, toothbrushing, interdental cleaning and dental attendance) and health conditions (body mass index [BMI], self‐rated health and history of chronic conditions and smoking) were included as covariates. Marital status was categorized as cohabiting (married and living with partner) or living alone (single, divorced or living apart and widowed). Smoking was determined based on four questions from the WHO (1998) questionnaire. Current smokers were those who reported that they smoked at least 100 times in their lifetime, they smoked regularly (daily for at least 1 year) and they last smoked today or the day before. Former smokers were those who reported that they smoked at least 100 times in their lifetime but did not smoke during the last year. Weekly alcohol intake (100% ethanol) was categorized as no use, moderate use (women < 70 g, men < 140 g) and risk use (women ≥ 70 g, men ≥ 140 g) (Shield et al. 2017). Physical activity was assessed with two questions on exercise during leisure time (LTE) for at least 30 min (so they felt at least slightly out of breath and sweating) and walking or cycling to work (WCW). Physical activity was categorized as ideal (LTE ≥ 4 times/week and WCW ≥ 30 min/day), sufficient (when only the LTE or WCW threshold was met), low (LTE = 2–3 times/week and WCW < 30 min/day) and sedentary (LTE ≤ 1 time/week and WCW < 30 min/day). Toothbrushing was reported using a 5‐point ordinal scale and categorized as twice or more daily, once daily or less than daily. The use of interdental cleaning aids (floss or interdental brushes) was reported using a 4‐point ordinal scale and categorized as daily, less than daily, or never. Dental attendance was reported using three response options and categorized as for check‐ups or only when in trouble (including those who had never visited the dentist). Participants' weight and height were measured by trained nurses using a wall‐mounted stadiometer and a bioimpedance device scale (InBody 3.0, Biospace, South Korea), respectively. Participants were classified as normal weight (BMI < 25 kg/m^2^), overweight (25–29.9 kg/m^2^) or obese (≥ 30 kg/m^2^). Current health status was reported on a 5‐point ordinal scale and categorized as poor, moderate or good. Participants also reported whether they had ever been diagnosed with diabetes, heart disease, hypertension or stroke.

### 2.3 Statistical Analysis

Descriptive analyses were performed in Stata MP 18 (StataCorp LP, College Station, Texas). We first compared the baseline characteristics of participants in each quintile of baseline starch intake (g/day) using the Chi‐square test. Then, we compared the NTPP in every wave (2000, 2004/2005 and 2011) by quintiles of baseline starch intake (expressed in g/day and %TEI, respectively). Tests for linear trend from crude negative binomial regression models were used for these comparisons.

The 11‐year change in the NTPP was modelled using mixed‐effects negative binomial regression, with the three repeated periodontal assessments (level 1) nested within individuals (level 2). Rate ratios (RRs) were reported as the measure of association. Time was modelled flexibly using a categorical indicator (coded as 0 for wave 1, 1 for wave 2 and 2 for wave 3), with both its intercept and slope incorporated as random effects to characterize individual differences in the baseline NTPP and the rate of change in NTPP over time. All other model predictors were estimated as fixed effects. The covariance matrix was estimated without any restrictions (unstructured). A model with time as the only predictor was initially fitted to determine the average annual rate of change in the NTPP. Then, the association between total starch intake at baseline and the 11‐year change in NTPP was evaluated in three sequential models. Model 1 included baseline starch intake, continuous EI and time as predictors. Model 2 further adjusted for sociodemographic factors, health behaviours and health condition. Model 3 also adjusted for the interaction between baseline starch intake and time. The deviance information criterion (DIC) was used to evaluate whether adding the interaction term improved the model fit (Spiegelhalter et al. 2002). As a rule of thumb, differences of more than 5 units rule out the model with the largest DIC. If the difference in the DIC is 5 units or less, the simpler model is preferred (Browne 2023; Spiegelhalter et al. 2002). The estimates from Model 2 represent the effect of baseline starch intake on the baseline NTPP score that will continue unchanged over the follow‐up period (parallel lines for the intake quintiles), whereas a significant interaction with time in Model 3 indicates that the effect of baseline starch intake on the NTPP score varies over time (divergent or convergent lines for the intake quintiles) (Singer and Willett 2003; Twisk 2013). Separate models were fitted for quintiles of baseline total starch intake in g/day and %EI, respectively. The same set of models was fitted for each of the eight food sources of starch separately. All models were fitted in MLwiN 3.08 (Centre for Multilevel Modelling, University of Bristol) using Markov Chain Monte Carlo (MCMC) algorithms, called from within Stata using the runmlwin command (Leckie and Charlton 2012).

We ran various supplemental analyses to evaluate the impact of some methodological choices on the findings. First, we tested the association between baseline starch intake and the number of teeth with progression in periodontal pocketing. Second, we used the number of teeth as an alternative outcome under the assumption that most teeth in adults are lost as a result of periodontal disease. Third, we tested the association between baseline starch intake and the NTPP among healthy participants (i.e., those with no history of chronic conditions such as hypertension, diabetes, stroke or heart disease). Fourth, we used a saturated model, which adjusts for all macronutrients (fat, protein, sugars, fibre and ethanol) instead of total energy intake, to evaluate the association between baseline starch intake (kcal) and the NTPP. Finally, we determined the power of our study to identify the observed associations.

## 3 Results

There were 1797 dentate adults who were periodontally examined in wave 1 and had participated in one or both follow‐up periodontal examinations. Of them, 428 were excluded because of missing information on starch intake (*n* = 86) or covariates (*n* = 342). The final sample included 1369 adults (623 men and 746 women), of whom 1207 had two waves of periodontal data and 162 had three waves of periodontal data. The mean age of participants was 47.4 ± 11.1 years at baseline.

The mean baseline starch intake was 130.0 ± 50.5 g/day, representing 23.1 ± 4.9 %EI. Male, older and less educated participants, those cohabiting and reporting no current smoking, no alcohol consumption and more physical activity were more likely to be in the highest quintiles of baseline starch intake (Table 1; Table S1 for men and Table S2 for women). The mean NTPP was 4.1 ± 5.6, 6.3 ± 5.6 and 4.8 ± 5.9 in waves 1, 2 and 3, respectively. There was a decreasing linear trend in the NTPP according to quintiles of baseline starch intake (g/day) in wave 1 (Table 2). Decreasing trends in the NTPP were also noted according to quintiles of baseline starch intake (%TEI) in wave 2.

**TABLE 1:** Description of participants according to quintiles of starch intake and baseline covariates.

Baseline covariates	All sample		*Q*1 (median: 71.3 g/day)		*Q*2 (median: 100.1 g/day)		*Q*3 (median: 123.2 g/day)		*Q*4 (median: 151.0 g/day)		*Q*5 (median: 197.0 g/day)	
	*n*	%	*n*	%	*n*	%	*n*	%	*n*	%	*n*	%
Sex												
Male	623	45.5	112	40.9	98	35.8	115	42.0	137	50	161	59.0
Female	746	54.5	162	59.1	176	67.2	159	58.0	137	50	112	41.0
Age groups												
30–39 years	406	29.7	83	30.3	82	29.9	83	30.3	83	30.3	75	27.5
40–49 years	405	29.6	78	28.5	78	28.5	84	30.7	83	30.3	82	30.0
50–59 years	348	25.4	85	31.0	76	27.7	58	21.2	64	23.4	65	23.8
60–69 years	168	12.3	27	9.9	28	10.2	38	13.9	35	12.8	40	14.7
70+ years	42	3.1	1	0.4	10	3.6	11	4.0	9	3.3	11	4.0
Education												
Basic	334	24.4	76	27.7	65	23.7	59	21.6	66	24.1	68	24.9
Secondary	507	37.0	91	33.2	84	30.7	93	33.9	110	40.1	129	47.3
Higher	528	38.6	107	39.1	125	45.6	122	44.5	98	35.8	76	27.8
Marital status												
Cohabiting	1037	75.8	184	67.2	191	69.7	216	78.8	219	79.9	227	83.2
Living alone	332	24.2	90	32.8	83	30.3	58	21.2	55	20.1	46	46.8
Smoking												
Never smoked	495	36.02	70	25.5	90	32.8	110	40.2	116	42.3	109	40.0
Former smokers	442	32.3	89	32.5	84	30.7	80	29.2	86	31.4	103	37.7
Current smokers	432	31.5	115	42.0	100	36.5	84	30.6	72	26.3	61	22.3
Alcohol consumption												
No use	143	10.4	12	4.40	19	6.9	26	9.5	32	11.7	54	19.8
Moderate use	918	67.1	174	63.5	181	66.1	193	70.4	191	69.7	179	65.6
Risk use	308	22.5	88	32.1	74	27.0	55	20.1	51	18.6	40	14.7
Physical activity												
Sedentary	523	38.2	135	49.3	97	35.4	98	35.8	98	35.8	95	34.8
Low	412	30.1	75	27.4	94	34.3	76	27.7	94	34.3	73	26.7
Sufficient	372	27.2	58	21.1	70	25.6	88	32.1	71	25.9	85	31.2
Ideal	62	4.50	6	2.20	13	4.7	12	4.40	11	4.00	20	7.3
BMI group												
Normal	562	41.1	111	40.5	112	44.5	120	43.8	98	35.8	111	40.7
Overweight	545	39.8	108	39.4	100	36.5	111	40.5	115	42.0	111	40.7
Obese	262	19.1	55	20.1	52	19.0	43	15.7	61	22.2	51	18.6
Diabetes												
No	1328	97.0	270	98.5	266	97.1	263	96.0	266	97.1	263	96.3
Yes	41	3.0	4	1.5	8	2.9	11	4.0	8	2.9	10	3.7
Heart disease												
No	1159	84.7	233	85.0	234	85.4	239	87.2	224	81.8	229	83.9
Yes	210	15.3	41	15.0	40	14.6	35	12.8	50	18.2	44	16.1
Hypertension												
No	1031	75.3	211	77.0	211	77.0	210	76.6	207	75.5	192	70.3
Yes	338	24.7	63	23.0	63	23.0	64	23.4	67	25.5	81	29.7
Stroke												
No	1349	98.5	271	98.9	270	98.5	273	99.6	269	98.2	266	97.4
Yes	20	1.5	3	1.1	4	1.5	1	0.4	5	1.8	7	2.6
Self‐rated general health												
Poor	66	4.9	13	4.7	12	4.4	12	4.4	14	5.1	15	5.5
Moderate	288	21.0	54	19.7	56	20.4	49	17.9	58	21.2	71	26.0
Good	1015	74.1	207	75.6	206	75.2	213	77.7	202	73.7	187	68.5
Toothbrushing												
Twice or more daily	933	68.1	189	69.0	198	72.3	187	68.2	191	69.7	168	61.6
Once daily	379	27.7	77	28.1	61	22.3	75	27.4	75	27.4	91	33.3
Less than daily	57	4.2	8	2.9	15	5.5	12	4.4	8	2.9	14	5.1
Interdental cleaning												
Daily	158	11.5	26	9.5	34	12.4	25	9.1	41	15.0	32	11.7
Less than daily	560	40.9	117	42.7	120	43.8	113	41.3	101	36.9	109	39.9
Never	651	47.6	131	47.8	120	43.8	136	49.6	132	48.1	132	48.4
Dental attendance												
For check‐ups	844	61.7	171	62.4	175	63.9	161	58.8	172	62.8	165	60.4
Only when in trouble	525	38.3	103	37.6	99	36.1	113	41.2	102	37.2	108	39.6

Abbreviations: BMI, body mass index; *Q*1–*Q*5, quintiles.

**TABLE 2:** Crude associations between total starch intake at baseline and PPD score in 2000, 2004 and 2011 (waves 1–3, respectively).

Total starch intake	Wave 1 (*n* = 1369)		Wave 2 (*n* = 704)		Wave 3 (*n* = 827)	
	Mean	(SD)	Mean	(SD)	Mean	(SD)
In g/day						
*Q*1 (71.3)	5.1	(6.3)	7.3	(6.1)	5.0	(6.1)
*Q*2 (100.7)	4.6	(6.0)	6.5	(5.9)	4.7	(6.2)
*Q*3 (123.2)	3.7	(5.4)	5.9	(5.7)	4.5	(5.5)
*Q*4 (151.0)	3.5	(5.2)	6.1	(5.6)	4.4	(4.8)
*Q*5 (197.0)	3.7	(4.9)	5.9	(4.9)	5.7	(6.6)
*p*‐value for trend^a^	0.002		0.082		0.646	
As %EI						
*Q*1 (17.2)	5.1	(6.5)	7.1	(6.0)	5.4	(6.6)
*Q*2 (20.7)	3.6	(5.2)	7.1	(5.9)	4.7	(5.6)
*Q*3 (22.9)	3.6	(5.3)	6.1	(5.3)	4.3	(5.6)
*Q*4 (25.4)	4.1	(5.5)	5.3	(5.8)	4.8	(5.9)
*Q*5 (29.4)	4.2	(5.3)	6.0	(5.1)	5.1	(5.7)
*p‐*value for trend^a^	0.295		0.019		0.711	

Abbreviations: SD, standard deviation; *Q*, quintiles.

^a^ Tests for linear trend were derived from negative binomial regression models.

In the mixed‐effects negative binomial regression models, there were baseline differences in the NTPP between quintiles of baseline starch intake (both in g/day and %EI), with adults in the third, fourth and fifth (highest) quintiles having lower NTPP at baseline than those in the first (lowest) quintile (Table 3). However, these associations were fully attenuated after adjustments for covariates. Adding the interaction between baseline starch intake and time did not improve the model fit (Table S3), suggesting that baseline starch intake was not associated with the change in the NTPP over time. Similar results were obtained in stratified analysis by sex (Table S4).

**TABLE 3:** The association between starch intake (expressed as g/day or %EI) and 11‐year change in the NTPP among Finnish adults ≥ 30 years (2900 observations in 1369 participants).

Starch intake	Model 1		Model 2	
	RR	(95% CI)	RR	(95% CI)
In g/day				
*Q*1 (71.3)		Reference		Reference
*Q*2 (100.7)	0.83	(0.68–1.01)	0.95	(0.77–1.14)
*Q*3 (123.2)	0.70	(0.57–0.86)	0.85	(0.68–1.04)
*Q*4 (151.0)	0.71	(0.56–0.89)	0.87	(0.68–1.08)
*Q*5 (197.0)	0.76	(0.55–0.99)	1.00	(0.75–1.35)
*p‐*value for trend		0.044		0.253
As %EI				
*Q*1 (17.2)		Reference		Reference
*Q*2 (20.7)	0.86	(0.70–1.04)	0.99	(0.80–1.19)
*Q*3 (22.9)	0.78	(0.64–0.94)	0.90	(0.74–1.10)
*Q*4 (25.4)	0.78	(0.65–0.95)	0.92	(0.74–1.11)
*Q*5 (29.4)	0.88	(0.72–1.07)	0.99	(0.80–1.20)
*p‐*value for trend		0.050		0.179

*Note*: Mixed‐effects negative binomial models were fitted to repeated measurements of the NTPP nested within participants and rate ratios (RRs) reported. Model 1 was adjusted for categorical time and continuous energy intake. Model 2 was additionally adjusted for sex, marital status, education, alcohol intake, physical activity, BMI group, history of hypertension, diabetes, heart disease and stroke, self‐rated general health, toothbrushing, interdental cleaning and dental attendance.

Abbreviations: %EI, percent of energy intake; NTTP, number of teeth with periodontal pocketing ≥ 4 mm; *Q*, quintiles.

In analysis by food groups, greater baseline intake of roots and tubers, pasta and wholegrains were associated with lower baseline NTPP (Table S5). Of those, only the baseline intake of wholegrains remained associated with the baseline NTPP after adjustments for covariates (Table 4). The model fit improved when adding the interaction between baseline wholegrains intake and time (Table S3). Compared to adults in the lowest quintile of baseline wholegrains intake, those in higher quintiles developed more teeth with periodontal pocketing during the follow‐up period. These findings indicate that the differences in the NTPP between quintiles of baseline wholegrains intake observed at baseline were reduced gradually over time.

**TABLE 4:** Association between wholegrains intake and 11‐year change in the NTPP among Finnish adults ≥ 30 years (2900 observations in 1369 participants).

	Model 1		Model 2		Model 3	
	RR	(95% CI)	RR	(95% CI)	RR	(95% CI)
Wholegrains (g/day)						
*Q*1 (median: 12.5)		Reference		Reference		Reference
*Q*2 (28.9)	0.78	(0.63–0.96)	0.89	(0.72–1.06)	0.74	(0.57–0.97)
*Q*3 (56.7)	0.94	(0.75–1.15)	1.00	(0.82–1.19)	1.03	(0.78–1.34)
*Q*4 (82.4)	0.73	(0.57–0.91)	0.80	(0.65–0.97)	0.68	(0.50–0.91)
*Q*5 (122.9)	0.80	(0.63–0.98)	0.84	(0.67–1.03)	0.78	(0.59–1.03)
*p‐*value for trend		0.011		0.035		0.061
Wholegrains × wave 2						
*Q*1 × wave 2						Reference
*Q*2 × wave 2					1.46	(0.97–2.06)
*Q*3 × wave 2					0.93	(0.65–1.32)
*Q*4 × wave 2					1.33	(0.89–1.81)
*Q*5 × wave 2					1.14	(0.77–1.63)
Wholegrains × wave 3						
*Q*1 × wave 3						Reference
*Q*2 × wave 3					1.25	(0.88–1.75)
*Q*3 × wave 3					0.98	(0.68–1.40)
*Q*4 × wave 3					1.30	(0.92–1.80)
*Q*5 × wave 3					1.16	(0.82–1.64)

*Note*: Mixed‐effects negative binomial models were fitted to repeated measurements of the NTPP nested within participants and rate ratios (RRs) reported. Model 1 was adjusted for categorical time and continuous energy intake. Model 2 was additionally adjusted for sex, marital status, education, alcohol intake, physical activity, BMI group, history of hypertension, diabetes, heart disease and stroke, self‐rated general health, toothbrushing, interdental cleaning and dental attendance. Model 3 further adjusted for the interaction between wholegrains intake quintiles and categorical time.

Abbreviations: NTTP, number of teeth with periodontal pocketing ≥ 4 mm; *Q*, quintiles.

The findings from various supplemental analyses confirmed our main findings. First, we found no association between baseline starch intake and the number of teeth with progression in periodontal pocketing (Table S6). Second, we found no association between baseline starch intake and the 11‐year change in number of teeth (Table S7). Third, no association was found among participants without history of chronic conditions (Table S8). Fourth, we found no association between baseline starch intake (kcal) and the NTPP in saturated models that adjusted for fat, protein, sugars, fibre and ethanol intake (Table S9). Finally, a power calculation indicated that our study had 87.5% power to reject the null hypothesis of no differences in the 11‐year change in the NTPP score observed between the highest (1.55 ± 6.93, *n* = 274) and lowest quintiles (−0.19 ± 6.11 teeth, *n* = 274) of baseline starch intake, using a two‐sided, two‐sample, unequal‐variance *t*‐test with a significance level of 0.05.

## 4 Discussion

This longitudinal study among Finnish adults found no association between baseline starch intake and changes in the NTPP over 11 years. Five of the six food groups high in starch were not associated with changes in the NTPP either. However, greater baseline intake of wholegrains was associated with lower NTPP at baseline and larger increases in the NTPP over time, suggesting a transient protective effect.

The association between starch intake and periodontitis has remained largely unexplored (Halvorsrud et al. 2019). Evidence from the medical field has been contradicting so far. Higher starch intake was associated with increased risk of diabetes, cardiovascular disease and mortality (Kanehara et al. 2021; Li et al. 2023). However, other studies have shown that starch intake was not associated with risk of cardiovascular events (AlEssa et al. 2018; Sieri et al. 2013). Although there are currently no dietary reference values for starch intake, the average intake of starch in our sample (g/day) was within the values reported across several adult populations in Nordic, Southern and Central European countries (Cust et al. 2009; Lemming and Pitsi 2022). This somewhat reduces the influence of the local food culture on our findings, at least in relation to total starch intake.

Our analysis by food groups showed that most common sources of starch evaluated here were not associated with changes in the NTPP. Potatoes, roots and tubers are considered RDS given their high glycaemic index (GI) and glycaemic load (GL) values. However, a recent meta‐analysis did not find an association between potato consumption and risk of all‐cause, cardiovascular and cancer mortality (Darooghegi Mofrad et al. 2020). Wholegrains, legumes and pasta are considered SDS given their low GI and GL values (Atkinson et al. 2021), and are part of the Mediterranean diet which can reduce systemic inflammation (Itsiopoulos, Mayr, and Thomas 2022). There is also evidence that the Mediterranean diet is associated with lower levels of gingival inflammation and lower odds of having periodontitis (Altun et al. 2021; Bartha et al. 2022; Wu et al. 2024). In the present study, we found a short‐term inverse dose–response relationship between the baseline consumption of wholegrains and the NTPP over time. That is, baseline differences in the NTPP favouring adults with higher wholegrains intake were fully reduced by wave 2 (after 4.5 years).

The above finding might reflect our study design, which included only a baseline dietary assessment. This longitudinal design provides a clear temporal ordering between exposure and outcome and control for baseline outcome and confounders. However, it assumes that a single dietary assessment suffices to characterize the long‐term dietary habits of participants. If this assumption does not hold, participants' intakes measured at baseline would substantially misclassify their true exposure during the study period (Willet 2013). In general, the stability of dietary intake data from FFQs decreases with time (Cui et al. 2021), which leads to reduced reproducibility and attenuation of diet–disease associations (Cade et al. 2002). In the Finnish context, a pooled analysis of three population‐based cohorts of adults, aged 25–70 years, showed some degree of stability in carbohydrate intake measured with the same FFQ used here. The absolute change in carbohydrate intake varied from −5.8 (SD = 89.3) to −55.7 (98.8) g/day over an average of 7 years, with 19%–24% of adults reporting < 10% change in intake over time (Tammi et al. 2023). The performance of the FFQ gives confidence in our dietary data to be used as long‐term exposure variables, at least between the first two waves (first 4.5 years of follow‐up). That said, our single dietary assessment could not capture variations in participants' intake over a decade. Therefore, it remains unknown whether changes in starch intake over the lifespan of individuals are associated with changes in the NTPP.

The strengths of this study include the relatively large sample, the multiple outcome assessments (three periodontal examinations over 11 years) and the comprehensive set of covariates used. Some limitations of the study are worth discussing, though. A first limitation is the moderate attrition between the baseline and follow‐up surveys. Retained participants were more likely to have more education, more favourable behaviours and better health. This means that caution must be exercised when generalizing the results. A second limitation was the use of periodontal pocketing as our study outcome, even though clinical attachment loss is the standard measure of disease progression. In addition, the examination of four instead of six periodontal sites and recording at the tooth level rather than at the site level reduced the variability in our study outcome. That said, our study is an improvement compared to the only previous longitudinal study which was based on self‐reported periodontitis. Also, similar findings were obtained when using the number of teeth with progression in periodontal pocketing and number of teeth as alternative outcomes in supplemental analysis.

The present findings have implications for future research before they can inform dietary recommendations to promote periodontal health. This novel research area would benefit from further evidence from observational (prospective) and intervention studies. Prospective studies of up to 5 years of follow‐up and high retention rates, in different populations, should incorporate annual dietary assessments to capture changes in starch intake, repeated full‐mouth periodontal examinations to estimate incidence and/or progression of periodontitis and control for time‐varying confounders. Future prospective studies could consider the classification of starch into RDS, SDS and RS. While this classification is currently challenging to operationalize in epidemiological studies (Halvorsrud et al. 2019), developing methods to incorporate these distinctions could provide more nuanced insights. Future prospective studies could also benefit from incorporating GI and GL values to differentiate between various starch sources. This approach could help disentangle the associations between different types of starches and periodontal health. Finally, our findings on wholegrains suggest that intervention studies should focus on specific starch sources rather than total starch intake. The intervention could target increasing the intake of wholegrains while decreasing the intake of other sources of starch while maintaining the same caloric intake (substitution models) to evaluate the effect of this substitution on periodontal status.

## 5 Conclusion

This study showed that greater starch intake at baseline was not associated with changes in the NTPP over 11 years. The intake of foods high in starch (potatoes, fried potatoes, roots and tubers, pasta and legumes) was not associated with changes in the NTPP either. That said, an inverse dose–response relationship between baseline wholegrains intake and the NTPP was found at baseline, although differences between intake quintiles were greatly reduced by the end of the follow‐up period.

## Author Contributions

Conceptualization: E.B., A.L.S. Data collection: A.L.S., A.L., S.M. Data curation: A.L.S., S.M. Data analysis: F.H.J., E.B., A.G. Writing – original draft: F.H.J., E.B. Writing – review and editing: F.H.J., A.L.S., A.L., S.M., A.G., E.B. All authors approved the final version of the manuscript submitted for consideration.

## Conflicts of Interest

The authors declare no conflicts of interest.

## Supporting information


Data S1.

## Data Availability

The data that support the findings of this study are available on request from the corresponding author. The data are not publicly available due to privacy or ethical restrictions.
