# Supplementary material for: Is Starch Intake Associated With Periodontal Status? An 11‐Year Longitudinal Analysis Among Finnish Adults
Source: J Clin Periodontol. 2024 Sep 24;52(3):353–62. doi: 10.1111/jcpe.14072 (PMC11830508; doi:10.1111/jcpe.14072)
Supplement: Supplementary file 1 — Data S1. [file JCPE-52-353-s001.docx]

**Table S1.** Description of the male participants according to quintiles of starch intake and baseline covariates (n=623)

| **Baseline covariates** | | **All men** | | **Q1** | | **Q2** | | **Q3** | | **Q4** | | **Q5** | |
| --- | --- | --- | --- | --- | --- | --- | --- | --- | --- | --- | --- | --- | --- |
|  |  | **n** | **%** | **n** | **%** | **n** | **%** | **n** | **%** | **n** | **%** | **n** | **%** |
| *Age groups* | |  |  |  |  |  |  |  |  |  |  |  |  |
|  | 30-39 years | 178 | 28.6 | 31 | 27.7 | 26 | 26.5 | 36 | 31.3 | 43 | 31.4 | 42 | 26.1 |
|  | 40-49 years | 176 | 28.3 | 34 | 30.4 | 25 | 25.5 | 29 | 25.2 | 36 | 26.3 | 52 | 28.3 |
|  | 50-59 years | 180 | 28.9 | 39 | 34.8 | 36 | 36.7 | 29 | 25.2 | 37 | 27.0 | 39 | 28.9 |
|  | 60-69 years | 70 | 11.2 | 8 | 7.1 | 7 | 7.1 | 16 | 13.9 | 17 | 12.4 | 22 | 11.2 |
|  | 70+ years | 19 | 3.1 | 0 | 0.0 | 4 | 4.1 | 5 | 4.4 | 4 | 2.9 | 6 | 3.1 |
| *Education* | |  |  |  |  |  |  |  |  |  |  |  |  |
|  | Basic | 132 | 21.2 | 26 | 23.2 | 20 | 23.7 | 21 | 18.3 | 31 | 22.6 | 34 | 21.1 |
|  | Secondary | 290 | 46.6 | 46 | 40.1 | 38 | 38.8 | 50 | 43.5 | 67 | 48.9 | 89 | 55.3 |
|  | Higher | 201 | 32.3 | 40 | 35.7 | 40 | 40.8 | 44 | 38.3 | 39 | 28.5 | 38 | 23.6 |
| *Marital status* | |  |  |  |  |  |  |  |  |  |  |  |  |
|  | Cohabiting | 501 | 80.4 | 80 | 71.4 | 71 | 72.4 | 98 | 85.2 | 115 | 83.9 | 137 | 85.1 |
|  | Living alone | 122 | 19.6 | 32 | 28.6 | 27 | 27.6 | 17 | 14.8 | 22 | 16.1 | 24 | 14.9 |
| *Smoking* | |  |  |  |  |  |  |  |  |  |  |  |  |
|  | Never smoked | 156 | 25.0 | 22 | 19.6 | 19 | 19.4 | 27 | 23.5 | 43 | 31.4 | 45 | 28.0 |
|  | Former smokers | 255 | 40.9 | 43 | 38.4 | 46 | 46.9 | 42 | 36.5 | 74 | 36.5 | 74 | 45.0 |
|  | Current smokers | 212 | 34.0 | 47 | 42.0 | 33 | 33.7 | 46 | 40.0 | 42 | 32.1 | 42 | 26.1 |
| *Alcohol consumption* | |  |  |  |  |  |  |  |  |  |  |  |  |
|  | No use | 56 | 9.0 | 4 | 3.6 | 3 | 3.1 | 5 | 4.4 | 13 | 9.5 | 31 | 19.3 |
|  | Moderate use | 390 | 62.6 | 59 | 52.7 | 60 | 61.2 | 77 | 67.0 | 90 | 65.7 | 104 | 64.6 |
|  | Risk use | 177 | 28.4 | 49 | 43.8 | 35 | 35.7 | 3 | 28.7 | 34 | 24.8 | 26 | 16.2 |
| *Physical activity* | |  |  |  |  |  |  |  |  |  |  |  |  |
|  | Sedentary | 243 | 39.0 | 63 | 56.3 | 32 | 32.7 | 37 | 32.2 | 54 | 39.4 | 57 | 35.4 |
|  | Low | 194 | 31.1 | 32 | 28.6 | 37 | 37.8 | 38 | 33.0 | 40 | 29.2 | 47 | 29.2 |
|  | Sufficient | 164 | 26.3 | 17 | 15.2 | 26 | 26.5 | 37 | 32.2 | 37 | 27.0 | 47 | 29.2 |
|  | Ideal | 22 | 3.5 | 0 | 0.0 | 3 | 3.1 | 3 | 2.6 | 6 | 4.4 | 10 | 6.2 |
| *BMI group* | |  |  |  |  |  |  |  |  |  |  |  |  |
|  | Normal | 208 | 33.4 | 32 | 28.6 | 36 | 36.8 | 40 | 34.8 | 37 | 27.0 | 63 | 39.1 |
|  | Overweight | 312 | 50.1 | 55 | 49.1 | 45 | 45.9 | 60 | 52.2 | 77 | 56.2 | 75 | 46.6 |
|  | Obese | 103 | 16.5 | 25 | 22.3 | 17 | 17.4 | 15 | 13.0 | 23 | 13.0 | 23 | 14.3 |
| *Diabetes* | |  |  |  |  |  |  |  |  |  |  |  |  |
|  | No | 604 | 97.0 | 111 | 99.1 | 96 | 98.0 | 110 | 95.7 | 134 | 97.8 | 153 | 95.0 |
|  | Yes | 19 | 3.0 | 1 | 0.9 | 2 | 2.0 | 5 | 4.4 | 3 | 2.2 | 8 | 5.0 |
| *Heart disease* | |  |  |  |  |  |  |  |  |  |  |  |  |
|  | No | 534 | 85.7 | 99 | 88.4 | 81 | 82.7 | 98 | 85.2 | 115 | 83.9 | 141 | 87.6 |
|  | Yes | 89 | 14.3 | 13 | 11.6 | 17 | 17.4 | 17 | 14.8 | 22 | 16.1 | 20 | 12.4 |
| *Hypertension* | |  |  |  |  |  |  |  |  |  |  |  |  |
|  | No | 468 | 75.1 | 87 | 77.7 | 74 | 75.5 | 88 | 76.5 | 106 | 77.4 | 113 | 70.2 |
|  | Yes | 155 | 24.9 | 25 | 22.3 | 24 | 24.5 | 27 | 23.5 | 31 | 22.6 | 48 | 29.8 |
| *Stroke* | |  |  |  |  |  |  |  |  |  |  |  |  |
|  | No | 612 | 98.2 | 110 | 98.2 | 96 | 98.0 | 114 | 99.1 | 136 | 99.3 | 156 | 96.9 |
|  | Yes | 11 | 1.8 | 2 | 1.8 | 2 | 2.0 | 1 | 0.9 | 1 | 0.7 | 5 | 3.1 |
| *Self-rated general health* | |  |  |  |  |  |  |  |  |  |  |  |  |
|  | Poor | 32 | 5.1 | 6 | 5.4 | 6 | 6.1 | 6 | 5.2 | 8 | 5.8 | 6 | 3.7 |
|  | Moderate | 130 | 20.9 | 19 | 17.0 | 21 | 21.4 | 21 | 18.3 | 31 | 22.6 | 38 | 23.6 |
|  | Good | 461 | 74.0 | 87 | 77.6 | 71 | 72.5 | 88 | 76.5 | 98 | 71.5 | 117 | 72.7 |
| *Toothbrushing* | |  |  |  |  |  |  |  |  |  |  |  |  |
|  | Twice or more daily | 326 | 52.3 | 64 | 57.1 | 48 | 49.0 | 55 | 47.8 | 80 | 58.4 | 79 | 49.1 |
|  | Once daily | 246 | 39.5 | 41 | 36.6 | 37 | 37.8 | 49 | 42.6 | 50 | 36.5 | 69 | 42.9 |
|  | Less than daily | 51 | 8.2 | 7 | 6.3 | 13 | 13.3 | 11 | 9.6 | 7 | 5.1 | 13 | 8.0 |
| *Interdental cleaning* | |  |  |  |  |  |  |  |  |  |  |  |  |
|  | Daily | 45 | 7.2 | 5 | 4.5 | 10 | 10.2 | 7 | 6.1 | 10 | 7.3 | 13 | 8.1 |
|  | Less than daily | 198 | 31.8 | 30 | 26.8 | 28 | 28.6 | 32 | 27.8 | 47 | 34.3 | 61 | 37.9 |
|  | Never | 380 | 61.0 | 77 | 68.7 | 60 | 61.2 | 76 | 66.1 | 80 | 58.4 | 87 | 54.0 |
| *Dental attendance* | |  |  |  |  |  |  |  |  |  |  |  |  |
|  | For check-ups | 342 | 54.9 | 56 | 50.0 | 55 | 56.1 | 50 | 43.5 | 80 | 58.4 | 101 | 62.7 |
|  | Only when in trouble | 281 | 45.1 | 56 | 50.0 | 43 | 43.9 | 65 | 56.5 | 57 | 41.6 | 60 | 37.3 |

BMI: Body mass index. Q: quintiles.

**Table S2.** Description of the female participants according to quintiles of starch intake and baseline covariates (n=746)

| **Baseline covariates** | | **All women** | | **Q1** | | **Q2** | | **Q3** | | **Q4** | | **Q5** | |
| --- | --- | --- | --- | --- | --- | --- | --- | --- | --- | --- | --- | --- | --- |
|  |  | **n** | **%** | **n** | **%** | **n** | **%** | **n** | **%** | **n** | **%** | **n** | **%** |
| *Age groups* | |  |  |  |  |  |  |  |  |  |  |  |  |
|  | 30-39 years | 228 | 30.6 | 52 | 32.1 | 56 | 31.8 | 47 | 29.6 | 40 | 29.2 | 33 | 29.5 |
|  | 40-49 years | 229 | 30.7 | 44 | 27.2 | 53 | 30.1 | 55 | 34.6 | 47 | 34.3 | 30 | 26.8 |
|  | 50-59 years | 168 | 22.5 | 46 | 28.4 | 40 | 22.7 | 29 | 18.2 | 27 | 19.7 | 26 | 23.2 |
|  | 60-69 years | 98 | 13.1 | 19 | 11.7 | 21 | 11.9 | 22 | 13.8 | 18 | 13.1 | 18 | 16.1 |
|  | 70+ years | 23 | 3.1 | 1 | 0.6 | 6 | 3.4 | 6 | 3.8 | 5 | 3.7 | 5 | 4.5 |
| *Education* | |  |  |  |  |  |  |  |  |  |  |  |  |
|  | Basic | 202 | 27.1 | 50 | 30.8 | 45 | 25.6 | 38 | 23.9 | 35 | 25.6 | 34 | 30.4 |
|  | Secondary | 217 | 29.1 | 45 | 27.8 | 46 | 26.1 | 43 | 27.0 | 43 | 31.4 | 40 | 35.7 |
|  | Higher | 327 | 43.8 | 67 | 41.4 | 85 | 48.3 | 78 | 49.1 | 59 | 43.0 | 38 | 33.9 |
| *Marital status* | |  |  |  |  |  |  |  |  |  |  |  |  |
|  | Cohabiting | 536 | 71.9 | 104 | 64.2 | 120 | 68.2 | 118 | 74.2 | 104 | 75.9 | 90 | 80.4 |
|  | Living alone | 210 | 28.1 | 58 | 35.8 | 56 | 31.8 | 41 | 25.8 | 33 | 24.1 | 22 | 19.6 |
| *Smoking* | |  |  |  |  |  |  |  |  |  |  |  |  |
|  | Never smoked | 339 | 45.4 | 48 | 29.6 | 71 | 40.3 | 83 | 52.2 | 73 | 53.3 | 64 | 57.1 |
|  | Former smokers | 187 | 25.1 | 46 | 28.4 | 38 | 21.6 | 38 | 23.9 | 36 | 26.3 | 29 | 25.9 |
|  | Current smokers | 220 | 29.5 | 68 | 42.0 | 67 | 38.1 | 38 | 23.9 | 28 | 20.4 | 19 | 17.0 |
| *Alcohol consumption* | |  |  |  |  |  |  |  |  |  |  |  |  |
|  | No use | 87 | 11.7 | 8 | 4.9 | 16 | 9.1 | 21 | 13.2 | 19 | 13.9 | 23 | 20.5 |
|  | Moderate use | 528 | 70.8 | 115 | 71.0 | 121 | 68.8 | 116 | 73.0 | 101 | 73.7 | 75 | 67.0 |
|  | Risk use | 131 | 17.6 | 39 | 24.1 | 39 | 22.2 | 22 | 13.8 | 17 | 12.4 | 14 | 12.5 |
| *Physical activity* | |  |  |  |  |  |  |  |  |  |  |  |  |
|  | Sedentary | 280 | 37.5 | 72 | 444.4 | 61 | 38.4 | 61 | 38.4 | 44 | 32.1 | 38 | 33.9 |
|  | Low | 218 | 29.2 | 43 | 26.5 | 38 | 23.9 | 38 | 23.9 | 54 | 39.4 | 26 | 23.2 |
|  | Sufficient | 208 | 27.9 | 41 | 25.3 | 51 | 32.1 | 51 | 32.1 | 34 | 24.8 | 38 | 33.9 |
|  | Ideal | 40 | 5.4 | 6 | 5.7 | 9 | 5.7 | 9 | 5.6 | 5 | 3.7 | 10 | 8.9 |
| *BMI group* | |  |  |  |  |  |  |  |  |  |  |  |  |
|  | Normal | 354 | 47.5 | 79 | 48.8 | 86 | 48.9 | 80 | 50.3 | 61 | 44.6 | 48 | 42.9 |
|  | Overweight | 233 | 31.2 | 53 | 32.7 | 55 | 31.2 | 51 | 32.1 | 38 | 27.7 | 36 | 32.1 |
|  | Obese | 159 | 21.3 | 30 | 18.5 | 35 | 19.9 | 28 | 17.6 | 38 | 27.7 | 28 | 25.0 |
| *Diabetes* | |  |  |  |  |  |  |  |  |  |  |  |  |
|  | No | 724 | 97.0 | 159 | 98.2 | 170 | 96.6 | 153 | 96.2 | 132 | 96.3 | 110 | 98.2 |
|  | Yes | 22 | 3.0 | 3 | 1.8 | 6 | 3.4 | 6 | 3.8 | 5 | 3.7 | 2 | 1.8 |
| *Heart disease* | |  |  |  |  |  |  |  |  |  |  |  |  |
|  | No | 625 | 83.8 | 134 | 82.7 | 153 | 86.9 | 141 | 88.7 | 109 | 79.6 | 88 | 78.6 |
|  | Yes | 121 | 16.2 | 28 | 17.3 | 23 | 13.1 | 18 | 11.3 | 28 | 20.4 | 24 | 21.4 |
| *Hypertension* | |  |  |  |  |  |  |  |  |  |  |  |  |
|  | No | 563 | 75.5 | 124 | 76.5 | 137 | 77.8 | 122 | 76.7 | 101 | 73.7 | 79 | 70.5 |
|  | Yes | 183 | 24.5 | 38 | 23.4 | 39 | 22.2 | 37 | 23.3 | 36 | 26.3 | 33 | 29.5 |
| *Stroke* | |  |  |  |  |  |  |  |  |  |  |  |  |
|  | No | 737 | 98.8 | 161 | 99.4 | 174 | 98.9 | 159 | 100 | 133 | 97.1 | 110 | 98.2 |
|  | Yes | 9 | 1.2 | 1 | 0.6 | 2 | 1.1 | 0 | 0 | 4 | 2.9 | 2 | 1.8 |
| *Self-rated general health* | |  |  |  |  |  |  |  |  |  |  |  |  |
|  | Poor | 34 | 4.5 | 7 | 4.3 | 6 | 3.4 | 6 | 3.8 | 6 | 4.4 | 9 | 8.0 |
|  | Moderate | 158 | 21.2 | 35 | 21.6 | 35 | 19.9 | 28 | 17.6 | 27 | 19.7 | 33 | 29.5 |
|  | Good | 554 | 74.3 | 120 | 74.1 | 135 | 76.7 | 125 | 78.6 | 104 | 75.9 | 70 | 62.5 |
| *Toothbrushing* | |  |  |  |  |  |  |  |  |  |  |  |  |
|  | Twice or more daily | 607 | 81.4 | 125 | 77.2 | 150 | 85.2 | 132 | 83.0 | 111 | 81.0 | 89 | 79.5 |
|  | Once daily | 133 | 17.8 | 36 | 22.2 | 24 | 13.6 | 26 | 16.4 | 25 | 18.3 | 22 | 19.6 |
|  | Less than daily | 6 | 0.8 | 1 | 0.6 | 2 | 1.1 | 1 | 0.6 | 1 | 0.7 | 1 | 0.9 |
| *Interdental cleaning* | |  |  |  |  |  |  |  |  |  |  |  |  |
|  | Daily | 113 | 15.2 | 21 | 13.0 | 24 | 13.6 | 18 | 11.3 | 31 | 22.6 | 19 | 17.0 |
|  | Less than daily | 362 | 48.5 | 87 | 53.7 | 92 | 52.3 | 81 | 51.0 | 54 | 39.4 | 48 | 42.9 |
|  | Never | 271 | 36.3 | 54 | 33.3 | 60 | 34.1 | 60 | 37.7 | 52 | 38.0 | 45 | 40.1 |
| *Dental attendance* | |  |  |  |  |  |  |  |  |  |  |  |  |
|  | For check-ups | 502 | 67.3 | 115 | 71.0 | 120 | 68.2 | 111 | 69.8 | 92 | 67.1 | 64 | 57.1 |
|  | Only when in trouble | 244 | 32.7 | 47 | 29.0 | 56 | 31.8 | 48 | 30.2 | 45 | 32.9 | 48 | 42.9 |

BMI: Body mass index. Q: quintiles.

**Table S3.** Comparison of goodness of fit statistics between models without and with the interaction between quintiles of baseline starch intake and time.

|  | | **Model without interaction** | **Model with interaction** | **Absolute difference** |
| --- | --- | --- | --- | --- |
| *Starch (g/day)* | |  |  |  |
|  | Deviance ($\bar{D}$) | 9992.86 | 9991.95 |  |
|  | Deviance ($\bar{\theta}$) | 8322.19 | 8324.62 |  |
|  | N of effective parameters (p_D_) | 1670.67 | 1667.33 |  |
|  | Bayesian DIC | 11663.52 | 11659.28 | -4.24 |
| *Starch (%EI)* | |  |  |  |
|  | Deviance ($\bar{D}$) | 9994.66 | 9996.64 |  |
|  | Deviance ($\bar{\theta}$) | 8321.35 | 8326.59 |  |
|  | N of effective parameters (p_D_) | 1673.32 | 1670.05 |  |
|  | Bayesian DIC | 11667.98 | 11666.69 | -1.29 |
| *Potatoes (g/day)* | |  |  |  |
|  | Deviance ($\bar{D}$) | 9990.34 | 10000.28 |  |
|  | Deviance ($\bar{\theta}$) | 8320.46 | 8328.24 |  |
|  | N of effective parameters (p_D_) | 1669.89 | 1672.04 |  |
|  | Bayesian DIC | 11660.23 | 11672.32 | 12.09 |
| *Potato products (g/day)* | |  |  |  |
|  | Deviance ($\bar{D}$) | 9993.38 | 9990.36 |  |
|  | Deviance ($\bar{\theta}$) | 8321.89 | 8320.84 |  |
|  | N of effective parameters (p_D_) | 1671.49 | 1669.52 |  |
|  | Bayesian DIC | 11664.87 | 11659.89 | -4.98 |
| *Roots and tubers (g/day)* | |  |  |  |
|  | Deviance ($\bar{D}$) | 9997.26 | 9994.54 |  |
|  | Deviance ($\bar{\theta}$) | 8325.37 | 8322.58 |  |
|  | N of effective parameters (p_D_) | 1671.89 | 1671.96 |  |
|  | Bayesian DIC | 11669.15 | 11666.49 | -2.66 |
| *Legumes (g/day)* | |  |  |  |
|  | Deviance ($\bar{D}$) | 9991.88 | 9989.14 |  |
|  | Deviance ($\bar{\theta}$) | 8321.49 | 8320.85 |  |
|  | N of effective parameters (p_D_) | 1670.38 | 1668.29 |  |
|  | Bayesian DIC | 11662.26 | 11657.44 | -4.82 |
| *Pasta (g/day)* | |  |  |  |
|  | Deviance ($\bar{D}$) | 9990.28 | 10000.42 |  |
|  | Deviance ($\bar{\theta}$) | 8319.50 | 8326.96 |  |
|  | N of effective parameters (p_D_) | 1670.78 | 1673.46 |  |
|  | Bayesian DIC | 11661.07 | 11673.89 | 12.82 |
| *Wholegrains (g/day)* | |  |  |  |
|  | Deviance ($\bar{D}$) | 9996.98 | 9993.97 |  |
|  | Deviance ($\bar{\theta}$) | 8324.99 | 8325.35 |  |
|  | N of effective parameters (p_D_) | 1671.99 | 1668.62 |  |
|  | Bayesian DIC | 11668.97 | 11662.59 | -6.38 |

**Table S4.** The association between baseline starch intake and 11-year change in the NTPP among male and female participants.

|  | | **Men (1326 observations in 623 participants)** | | | | **Women (1574 observations in 746 participants)** | | | |
| --- | --- | --- | --- | --- | --- | --- | --- | --- | --- |
|  |  | **Model 1** | | **Model 2** | | **Model 1** | | **Model 2** | |
|  |  | **RR** | **(95% CI)** | **RR** | **(95% CI)** | **RR** | **(95% CI)** | **RR** | **(95% CI)** |
| *Starch (g/day)* | |  |  |  |  |  |  |  |  |
|  | Q1 (lowest) |  | Reference |  | Reference |  | Reference |  | Reference |
|  | Q2 | 0.93 | (0.69, 1.20) | 0.99 | (0.74, 1.26) | 0.85 | (0.63, 1.12) | 0.88 | (0.64, 1.16) |
|  | Q3 | 0.77 | (0.57, 1.01) | 0.83 | (0.62, 1.07) | 0.73 | (0.52, 1.01) | 0.81 | (0.56, 1.16) |
|  | Q4 | 0.79 | (0.56, 1.07) | 0.92 | (0.67, 1.25) | 0.71 | (0.48, 1.03) | 0.81 | (0.52, 1.15) |
|  | Q5 (highest) | 0.95 | (0.61, 1.36) | 1.21 | (0.77, 1.80) | 0.70 | (0.43, 1.11) | 0.83 | (0.46, 1.28) |
|  | *P value for trend* |  | *0.115* |  | *0.369* |  | *0.037* |  | *0.143* |
| *Starch (%EI)* | |  |  |  |  |  |  |  |  |
|  | Q1 (lowest) |  | Reference |  | Reference |  | Reference |  | Reference |
|  | Q2 | 0.98 | (0.76, 1.25) | 1.15 | (0.90, 1.45) | 0.78 | (0.57, 1.03) | 0.81 | (0.59, 1.08) |
|  | Q3 | 0.92 | (0.71, 1.18) | 0.99 | (0.78, 1.25) | 0.70 | (0.51, 0.92) | 0.76 | (0.55, 1.02) |
|  | Q4 | 0.93 | (0.71, 1.19) | 1.06 | (0.83, 1.35) | 0.68 | (0.50, 0.90) | 0.78 | (0.57, 1.04) |
|  | Q5 (highest) | 0.96 | (0.75, 1.23) | 1.10 | (0.85, 1.41) | 0.79 | (0.58, 1.05) | 0.87 | (0.62, 1.18) |
|  | *P value for trend* |  | *0.277* |  | *0.338* |  | *0.034* |  | *0.144* |
| *Potatoes (g/day)* | |  |  |  |  |  |  |  |  |
|  | Q1 (lowest) |  | Reference |  | Reference |  | Reference |  | Reference |
|  | Q2 | 1.14 | (0.89, 1.48) | 1.11 | (0.88, 1.40) | 0.91 | (0.68, 1.20) | 0.88 | (0.63, 1.17) |
|  | Q3 | 1.07 | (0.82, 1.38) | 1.03 | (0.80, 1.32) | 0.82 | (0.61, 1.06) | 0.84 | (0.61, 1.11) |
|  | Q4 | 0.98 | (0.76, 1.27) | 0.90 | (0.70, 1.15) | 0.76 | (0.57, 1.01) | 0.80 | (0.59, 1.09) |
|  | Q5 (highest) | 0.98 | (0.73, 1.33) | 0.94 | (0.72, 1.23) | 1.01 | (0.72, 1.37) | 0.93 | (0.64, 1.30) |
|  | *P value for trend* |  | *0.192* |  | *0.132* |  | *0.161* |  | *0.254* |
| *Potato products (g/day)* | |  |  |  |  |  |  |  |  |
|  | Q1 (lowest) |  | Reference |  | Reference |  | Reference |  | Reference |
|  | Q2 | 0.81 | (0.59, 1.07) | 0.83 | (0.60, 1.14) | 0.97 | (0.73, 1.26) | 0.98 | (0.75, 1.27) |
|  | Q3 | 0.67 | (0.50, 0.86) | 0.69 | (0.49, 0.94) | 0.98 | (0.74, 1.28) | 0.96 | (0.72, 1.25) |
|  | Q4 | 0.84 | (0.65, 1.09) | 0.86 | (0.62, 1.17) | 0.94 | (0.69, 1.26) | 1.01 | (0.72, 1.38) |
|  | Q5 (highest) | 0.88 | (0.67, 1.13) | 0.90 | (0.64, 1.26) | 0.98 | (0.69, 1.34) | 1.06 | (0.73, 1.50) |
|  | *P value for trend* |  | *0.351* |  | *0.405* |  | *0.355* |  | *0.447* |
| *Roots and tubers (g/day)* | |  |  |  |  |  |  |  |  |
|  | Q1 (lowest) |  | Reference |  | Reference |  | Reference |  | Reference |
|  | Q2 | 1.20 | (0.95, 1.50) | 1.25 | (0.99, 1.54) | 0.94 | (0.68, 1.28) | 0.93 | (0.68, 1.23) |
|  | Q3 | 1.05 | (0.83, 1.31) | 1.11 | (0.88, 1.39) | 0.80 | (0.57 1.06) | 0.79 | (0.58, 1.07) |
|  | Q4 | 0.95 | (0.73, 1.21) | 1.01 | (0.77, 1.28) | 0.95 | (0.68, 1.29) | 0.95 | (0.68, 1.28) |
|  | Q5 (highest) | 1.15 | (0.88, 1.48) | 1.19 | (0.90, 1.58) | 0.96 | (0.69, 1.28) | 0.89 | (0.64, 1.22) |
|  | *P value for trend* |  | *0.467* |  | *0.266* |  | *0.454* |  | *0.311* |
| *Pasta (g/day)* | |  |  |  |  |  |  |  |  |
|  | Q1 (lowest) |  | Reference |  | Reference |  | Reference |  | Reference |
|  | Q2 | 1.05 | (0.81, 1.33) | 1.00 | (0.77, 1.28) | 1.15 | (0.86, 1,56) | 1.16 | (0.88, 1.520 |
|  | Q3 | 1.25 | (0.96, 1.60) | 1.18 | (0.91, 1.48) | 0.99 | (0.74, 1.35) | 1.02 | (0.75, 1.37) |
|  | Q4 | 1.11 | (0.88, 1.38) | 1.22 | (0.95, 1,54) | 0.78 | (0.57, 1.05) | 0.79 | (0.58, 1.08) |
|  | Q5 (highest) | 1.03 | (0.78, 1.32) | 1.10 | (0.82, 1.44) | 0.76 | (0.56, 1.03) | 0.90 | (0.64, 1.23) |
|  | *P value for trend* |  | *0.316* |  | *0.114* |  | *0.003* |  | *0.067* |
| *Legumes (g/day)* | |  |  |  |  |  |  |  |  |
|  | Q1 (lowest) |  | Reference |  | Reference |  | Reference |  | Reference |
|  | Q2 | 0.98 | (0.75, 1.27) | 0.99 | (0.75, 1.28) | 1.04 | (0.78, 1.37) | 1.04 | (0.80, 1.34) |
|  | Q3 | 0.96 | (0.74, 1.26) | 1.01 | (0.77, 1.30) | 0.65 | (0.47, 0.88) | 0.64 | (0.47, 0.86) |
|  | Q4 | 1.15 | (0.86, 1.49) | 1.19 | (0.89, 1.54) | 0.97 | (0.70, 1.31) | 0.91 | (0.66, 1.22) |
|  | Q5 (highest) | 1.11 | (0.83, 1.43) | 1.13 | (0.84, 1.50) | 1.01 | (0.74, 1.37) | 0.98 | (0.72, 1.32) |
|  | *P value for trend* |  | *0.147* |  | *0.061* |  | *0.366* |  | *0.192* |
| *Wholegrains (g/day)* | |  |  |  |  |  |  |  |  |
|  | Q1 (lowest) |  | Reference |  | Reference |  | Reference |  | Reference |
|  | Q2 | 0.83 | (0.64, 1.08) | 0.91 | (0.70, 1.16) | 0.80 | (0.59, 1.08) | 0.87 | (0.62, 1.18) |
|  | Q3 | 1.00 | (0.78, 1.27) | 1.04 | (0.82, 1.33) | 0.97 | (0.71, 1.30) | 1.01 | (0.74, 1.35) |
|  | Q4 | 0.78 | (0.60, 1.00) | 0.85 | (0.65, 1.12) | 0.74 | (0.53, 1.01) | 0.74 | (0.52, 1.01) |
|  | Q5 (highest) | 0.86 | (0.65, 110) | 0.89 | (0.69, 1.17) | 0.78 | (0.55, 1.08) | 0.85 | (0.60, 1.19) |
|  | *P value for trend* |  | *0.161* |  | *0.185* |  | *0.073* |  | *0.058* |

NTTP: Number of teeth with periodontal pocketing>4mm, Q: quintiles; %EI: percent of energy intake.

Mixed-effects negative binomial models were fitted to repeated measurements of the NTPP nested within participants. Rate ratios (RR) were reported. Model 1 was adjusted for categorical time and continuous energy intake. Model 2 was additionally adjusted for sex, marital status, education, alcohol intake, physical activity, BMI group, history of hypertension, diabetes, heart disease and stroke, self-rated general health, toothbrushing, interdental cleaning and dental attendance.

**Table S5.** The association between baseline intake of selected groups high in starch and 11-year change in the NTPP among Finnish adults >30 years (2900 observations in 1369 participants).

| **Food groups** | | **Model 1** | | **Model 2** | |
| --- | --- | --- | --- | --- | --- |
|  |  | **RR** | **(95% CI)** | **RR** | **(95% CI)** |
| *Potatoes (g/day)* | |  |  |  |  |
|  | Q1 (median: 66.4) |  | Reference |  | Reference |
|  | Q2 (104.0) | 1.01 | (0.83, 1.24) | 0.99 | (0.83, 1.19) |
|  | Q3 (140.6) | 0.90 | (0.74, 1.12) | 0.93 | (0.76, 1.12) |
|  | Q4 (181.8) | 0.84 | (0.69, 1.02) | 0.87 | (0.71, 1.10) |
|  | Q5 (247.3) | 0.95 | (0.75, 1.20) | 0.95 | (0.77, 1.16) |
|  | *P value for trend* |  | *0.073* |  | *0.100* |
| *Potato products (g/day)* | |  |  |  |  |
|  | Q1 (median: 0.8) |  | Reference |  | Reference |
|  | Q2 (3.4) | 0.91 | (0.75, 1.11) | 0.93 | (0.76, 1.13) |
|  | Q3 (4.6) | 0.87 | (0.71, 1.06) | 0.85 | (0.69, 1.02) |
|  | Q4 (6.2) | 1.01 | (0.81, 1.25) | 0.95 | (0.77, 1.16) |
|  | Q5 (9.5) | 1.12 | (0.90, 1.38) | 1.01 | (0.81, 1.21) |
|  | *P value for trend* |  | *0.209* |  | *0.500* |
| *Roots and tubers (g/day)* | |  |  |  |  |
|  | Q1 (median: 13.5) |  | Reference |  | Reference |
|  | Q2 (24.7) | 1.03 | (0.85, 1.23) | 1.12 | (0.99, 1.36) |
|  | Q3 (40.8) | 0.90 | (0.73, 1.10) | 0.95 | (0.78, 1.18) |
|  | Q4 (60.6) | 0.87 | (0.70, 1.07) | 1.03 | (0.82, 1.25) |
|  | Q5 (93.1) | 0.88 | (0.72, 1.08) | 1.05 | (0.83, 1.32) |
|  | *P value for trend* |  | *0.019* |  | *0.468* |
| *Pasta (g/day)* | |  |  |  |  |
|  | Q1 (median: 2.9) |  | Reference |  | Reference |
|  | Q2 (3.0) | 1.03 | (0.84, 1.26) | 1.11 | (0.91, 1.32) |
|  | Q3 (6.3) | 1.07 | (0.88, 1.31) | 1.12 | (0.92, 1.34) |
|  | Q4 (6.4) | 0.91 | (0.76, 1.09) | 0.99 | (0.83, 1.19) |
|  | Q5 (19.1) | 0.82 | (0.67, 0.99) | 1.00 | (0.80, 1.22) |
|  | *P value for trend* |  | *0.026* |  | *0.374* |
| *Legumes (g/day)* | |  |  |  |  |
|  | Q1 (median: 2.8) |  | Reference |  | Reference |
|  | Q2 (6.9) | 1.01 | (0.82, 1.22) | 1.02 | (0.83, 1.21) |
|  | Q3 (9.9) | 0.82 | (0.65, 0.99) | 080 | (0.66, 0.97) |
|  | Q4 (14.7) | 1.09 | (0.88, 1.32) | 1.05 | (0.86, 1.27) |
|  | Q5 (33.7) | 1.02 | (0.81, 1.25) | 1.03 | (0.82, 1.26) |
|  | *P value for trend* |  | *0.300* |  | *0.461* |

NTTP: Number of teeth with periodontal pocketing>4mm, Q: quintiles.

Mixed-effects negative binomial models were fitted to repeated measurements of the NTPP nested within participants. Rate ratios (RR) were reported. Model 1 was adjusted for categorical time and continuous energy intake. Model 2 was additionally adjusted for sex, marital status, education, alcohol intake, physical activity, BMI group, history of hypertension, diabetes, heart disease and stroke, self-rated general health, toothbrushing, interdental cleaning and dental attendance.

**Table S6.** The association between baseline starch intake and number of teeth with progression in periodontal pocketing over 11 years among Finnish adults >30 years (1369 participants).

|  | | | **Model 1** | | **Model 2** | |
| --- | --- | --- | --- | --- | --- | --- |
|  |  |  | **Coef.** | **(95% CI)** | **Coef.** | **(95% CI)** |
| *Starch (g/day)* | | |  |  |  |  |
|  | Q1 (median: 71.3) | |  | Reference |  | Reference |
|  | Q2 (100.7) | | -0.76 | (-2.85, 1.33) | -0.17 | (-2.26, 1.92) |
|  | Q3 (123.2) | | -0.47 | (-2.68, 1.73) | 0.15 | (-2.10, 2.40) |
|  | Q4 (151.0) | | -1.44 | (-3.86, 0.98) | -0.41 | (-2.89, 2.07) |
|  | Q5 (197.0) | | -1.22 | (-4.25, 1.80) | 0.23 | (-2.93, 3.39) |
|  | *P value for trend* | |  | *0.347* |  | *0.983* |
| *Starch (%EI)* | | |  |  |  |  |
|  | Q1 (median: 17.2) | |  | Reference |  | Reference |
|  | Q2 (20.7) | | 0.42 | (-1.62, 2.46) | 0.84 | (-1.21, 2.90) |
|  | Q3 (22.9) | | 0.83 | (-1.21, 2.87) | 1.17 | (-0.88, 3.23) |
|  | Q4 (25.4) | | -0.38 | (-2.41, 1.66) | 0.29 | (-1.78, 2.37) |
|  | Q5 (29.4) | | -0.72 | (-2.76, 1.31) | 0.44 | (-1.70, 2.59) |
|  | *P value for trend* | |  | *0.335* |  | *0.874* |
| *Potatoes (g/day)* | | |  |  |  |  |
|  | | Q1 (median: 66.4) |  | Reference |  | Reference |
|  | | Q2 (104.0) | 2.62 | (0.58, 4.66) | 2.79 | (0.75, 4.83) |
|  | | Q3 (140.6) | 4.24 | (2.17, 6.32) | 4.55 | (2.47, 6.63) |
|  | | Q4 (181.8) | 3.30 | (1.22, 5.38) | 3.79 | (1.67, 5.90) |
|  | | Q5 (247.3) | 4.88 | (2.60, 7.17) | 5.34 | (3.02, 7.66) |
|  | | *P value for trend* |  | *<0.001* |  | *<0.001* |
| *Potato products (g/day)* | | |  |  |  |  |
|  | | Q1 (median: 0.8) |  | Reference |  | Reference |
|  | | Q2 (3.4) | 0.26 | (-1.77, 2.29) | -0.06 | (-2.12, 2.01) |
|  | | Q3 (4.6) | 0.22 | (-1.83, 2.27) | -0.23 | (-2.34, 1.87) |
|  | | Q4 (6.2) | 2.44 | (0.32, 4.56) | 1.59 | (-0.62, 3.81) |
|  | | Q5 (9.5) | 2.73 | (0.56, 4.89) | 1.49 | (-0.83, 3.80) |
|  | | *P value for trend* |  | *0.003* |  | *0.088* |
| *Roots and tubers (g/day)* | | |  |  |  |  |
|  | | Q1 (median: 13.5) |  | Reference |  | Reference |
|  | | Q2 (24.7) | 2.44 | (0.39, 4.48) | 3.15 | (1.11, 5.18) |
|  | | Q3 (40.8) | 1.54 | (-0.51, 3.59) | 2.59 | (0.52, 4.65) |
|  | | Q4 (60.6) | 1.80 | (-0.30, 3.90) | 3.12 | (0.98, 5.25) |
|  | | Q5 (93.1) | 0.88 | (-1.30, 3.06) | 2.30 | (0.02, 4.58) |
|  | | *P value for trend* |  | *0.655* |  | *0.080* |
| *Pasta (g/day)* | | |  |  |  |  |
|  | | Q1 (median: 2.9) |  | Reference |  | Reference |
|  | | Q2 (3.0) | 1.76 | (-0.24, 3.76) | 1.14 | (-0.89, 3.16) |
|  | | Q3 (6.3) | 0.56 | (-1.46, 2.58) | 0.00 | (-2.05, 2,04) |
|  | | Q4 (6.4) | 2.23 | (0.32, 4.15) | 1.23 | (-0.81, 3.28) |
|  | | Q5 (19.1) | 0.20 | (-1.82, 2.21) | -0.56 | (-2.79, 1.66) |
|  | | *P value for trend* |  | *0.478* |  | *0.850* |
| *Legumes (g/day)* | | |  |  |  |  |
|  | Q1 (median: 2.8) | |  | Reference |  | Reference |
|  | Q2 (6.9) | | 0.18 | (-1.81, 2.18) | 0.39 | (-1.60, 2.38) |
|  | Q3 (9.9) | | 0.03 | (-2.07, 2.14) | 0.48 | (-1.62, 2.58) |
|  | Q4 (14.7) | | 1.19 | (-0.91, 3.29) | 1.47 | (-0.63, 3.56) |
|  | Q5 (33.7) | | 0.18 | (-1.98, 2.34) | 0.89 | (-1.28, 3.05) |
|  | *P value for trend* | |  | *0.580* |  | *0.253* |
| *Wholegrains (g/day)* | | |  |  |  |  |
|  | Q1 (median: 12.5) | |  | Reference |  | Reference |
|  | Q2 (28.9) | | -1.57 | (-3.62, 0.49) | -1.13 | (-3.18, 0.92) |
|  | Q3 (56.7) | | -1.17 | (-3.22, 0.88) | -0.47 | (-2.55, 1.62) |
|  | Q4 (82.4) | | -2.67 | (-4.79, -0.55) | -1.58 | (-3.77, 0.61) |
|  | Q5 (122.9) | | -2.18 | (-4.34, -0.01) | -1.39 | (-3.62, 0.85) |
|  | *P value for trend* | |  | *0.032* |  | *0.234* |

Q: quintiles; %EI: percent of energy intake.

Linear regression models were fitted to the number of teeth with progression in periodontal pocketing over 11 years. Unstandardised regression coefficients (Coef.) were reported. Model 1 was adjusted for continuous energy intake. Model 2 was additionally adjusted for sex, marital status, education, alcohol intake, physical activity, BMI group, history of hypertension, diabetes, heart disease and stroke, self-rated general health, toothbrushing, interdental cleaning and dental attendance.

**Table S7.** The association between baseline starch intake and 11-year change in number of teeth among Finnish adults >30 years (2900 observations in 1369 participants).

|  | | | **Model 1** | | **Model 2** | |
| --- | --- | --- | --- | --- | --- | --- |
|  |  |  | **Coef.** | **(95% CI)** | **Coef.** | **(95% CI)** |
| *Starch (g/day)* | | |  |  |  |  |
|  | Q1 (71.3) | |  | Reference |  | Reference |
|  | Q2 (100.7) | | -0.70 | (-1.87, 0.47) | -0.29 | (-1.22, 0.64) |
|  | Q3 (123.2) | | -1.34 | (-2.57, -0.10) | -0.56 | (-1.57, 0.44) |
|  | Q4 (151.0) | | -2.00 | (-3.36, -0.65) | -0.54 | (-1.65, 0.56) |
|  | Q5 (197.0) | | -3.23 | (-4.92, -1.53) | -0.44 | (-1.85, 0.97) |
|  | *P value for trend* | |  | *<0.001* |  | *0.412* |
| *Starch (%EI)* | | |  |  |  |  |
|  | Q1 (17.2) | |  | Reference |  | Reference |
|  | Q2 (20.7) | | 0.86 | (-0.27, 2.00) | 0.07 | (-0.84, 0.99) |
|  | Q3 (22.9) | | 0.08 | (-1.06, 1.21) | 0.15 | (-0.77, 1.06) |
|  | Q4 (25.4) | | -1.08 | (-2.21, 0.05) | -0.21 | (-1.14, 0.71) |
|  | Q5 (29.4) | | -2.43 | (-3.57, -1.30) | -0.39 | (-1.35, 0.56) |
|  | *P value for trend* | |  | *<0.001* |  | *0.340* |
| *Potatoes (g/day)* | | |  |  |  |  |
|  | | Q1 (median: 66.4) |  | Reference |  | Reference |
|  | | Q2 (104.0) | -1.28 | (-2.43, -0.13) | -0.39 | (-1.31, 0.53) |
|  | | Q3 (140.6) | -1.22 | (-2.39, -0.05) | -0.28 | (-1.22, 0.66) |
|  | | Q4 (181.8) | -2.00 | (-3.17, -0.83) | -0.10 | (-1.06, 0.85) |
|  | | Q5 (247.3) | -3.16 | (-4.45, -1.87) | -0.53 | (-1.57, 0.52) |
|  | | *P value for trend* |  | *<0.001* |  | *0.567* |
| *Potato products (g/day)* | | |  |  |  |  |
|  | | Q1 (median: 0.8) |  | Reference |  | Reference |
|  | | Q2 (3.4) | 1.13 | (0.00, 2.26) | -0.40 | (-1.32, 0.52) |
|  | | Q3 (4.6) | 1.20 | (0.06, 2.35) | -0.51 | (-1.45, 0.43) |
|  | | Q4 (6.2) | 2.10 | (0.91, 3.29) | -0.25 | (-1.24, 0.73) |
|  | | Q5 (9.5) | 3.24 | (2.03, 4.45) | 0.50 | (-0.54, 1.53) |
|  | | *P value for trend* |  | *<0.001* |  | *0.329* |
| *Roots and tubers (g/day)* | | |  |  |  |  |
|  | | Q1 (median: 13.5) |  | Reference |  | Reference |
|  | | Q2 (24.7) | 0.53 | (-0.62, 1.68) | 0.43 | (-0.49, 1.34) |
|  | | Q3 (40.8) | -0.45 | (-1.61, 0.70) | 0.17 | (-0.75, 1.10) |
|  | | Q4 (60.6) | 0.43 | (-0.75, 1.61) | 0.68 | (-0.27, 1.63) |
|  | | Q5 (93.1) | -1.02 | (-2.25, 0.20) | 0.30 | (-0.72, 1.32) |
|  | | *P value for trend* |  | *0.128* |  | *0.455* |
| *Pasta (g/day)* | | |  |  |  |  |
|  | | Q1 (median: 2.9) |  | Reference |  | Reference |
|  | | Q2 (3.0) | 1.16 | (0.07, 2.25) | 0.20 | (-0.71, 1.10) |
|  | | Q3 (6.3) | 2.15 | (1.06, 3.25) | 0.51 | (-0.40, 1.43) |
|  | | Q4 (6.4) | 4.08 | (3.04, 5.13) | 0.71 | (-0.20, 1.63) |
|  | | Q5 (19.1) | 5.07 | (3.98, 6.17) | 0.73 | (-0.26, 1.73) |
|  | | *P value for trend* |  | *<0.001* |  | *0.081* |
| *Legumes (g/day)* | | |  |  |  |  |
|  | Q1 (median: 2.8) | |  | Reference |  | Reference |
|  | Q2 (6.9) | | 0.27 | (-0.86, 1.39) | 0.13 | (-0.76, 1.02) |
|  | Q3 (9.9) | | -0.01 | (-1.19, 1.18) | -0.43 | (-1.37, 0.51) |
|  | Q4 (14.7) | | 0.95 | (-0.23, 2.13) | 0.37 | (-0.56, 1.31) |
|  | Q5 (33.7) | | 0.74 | (-0.48, 1.95) | 0.48 | (-0.49, 1.45) |
|  | *P value for trend* | |  | *0.126* |  | *0.294* |
| *Wholegrains (g/day)* | | |  |  |  |  |
|  | Q1 (median: 12.5) | |  | Reference |  | Reference |
|  | Q2 (28.9) | | -1.32 | (-2.46, -0.18) | -1.01 | (-1.92, -0.10) |
|  | Q3 (56.7) | | -0.92 | (-2.06, 0.22) | 0.30 | (-0.62, 1.23) |
|  | Q4 (82.4) | | -3.65 | (-4.83, -2.47) | -1.39 | (-2.36, -0.43) |
|  | Q5 (122.9) | | -2.59 | (-3.80, -1.39) | -0.56 | (-1.55, 0.43) |
|  | *P value for trend* | |  | *<0.001* |  | *0.263* |

Q: quintiles; %EI: percent of energy intake.

Linear mixed-effects models were fitted to repeated measurements of the number of teeth nested within participants. Unstandardised regression coefficients (Coef.) were reported. Model 1 was adjusted for categorical time and continuous energy intake. Model 2 was additionally adjusted for sex, marital status, education, alcohol intake, physical activity, BMI group, history of hypertension, diabetes, heart disease and stroke, self-rated general health, toothbrushing, interdental cleaning and dental attendance.

**Table S8.** The association between baseline starch intake and11-year change in the NTPP among Finnish adults >30 years with no chronic conditions (1895 observations in 887 participants).

|  | | | | **Model 1** | | **Model 2** | |
| --- | --- | --- | --- | --- | --- | --- | --- |
|  |  |  |  | **RR** | **(95% CI)** | **RR** | **(95% CI)** |
| *Starch (g/day)* | | | |  |  |  |  |
|  | | | Q1 (lowest) |  | Reference |  | Reference |
|  | | | Q2 | 0.80 | (0.61, 1.02) | 0.96 | (0.74, 1.21) |
|  | | | Q3 | 0.69 | (0.53, 0.88) | 0.84 | (0.64, 1.07) |
|  | | | Q4 | 0.72 | (0.54, 0.96) | 0.87 | (0.63, 1.15) |
|  | | | Q5 (highest) | 0.87 | (0.60, 1.29) | 1.11 | (0.75, 1.55) |
|  | | | *P value for trend* |  | *0.047* |  | *0.354* |
| *Starch (%EI)* | | | |  |  |  |  |
|  | | | Q1 (lowest) |  | Reference |  | Reference |
|  | | | Q2 | 0.77 | (0.59, 0.98) | 0.90 | (0.71, 1.12) |
|  | | | Q3 | 0.69 | (0.53, 0.88) | 0.77 | (0.60, 0.97) |
|  | | | Q4 | 0.79 | (0.61, 1.00) | 0.91 | (0.71, 1.14) |
|  | | | Q5 (highest) | 0.92 | (0.70, 1.19) | 1.06 | (0.82, 1.34) |
|  | | | *P value for trend* |  | *0.246* |  | *0.487* |
| *Potatoes (g/day)* | | | |  |  |  |  |
|  | | Q1 (lowest) | |  | Reference |  | Reference |
|  | | Q2 | | 1.00 | (0.78, 1,27) | 0.97 | (0.77, 1.23) |
|  | | Q3 | | 0.97 | (0.75, 1.22) | 0.99 | (0.77, 1.26) |
|  | | Q4 | | 0.88 | (0.67, 1.11) | 0.90 | (0.69, 1.16) |
|  | | Q5 (highest) | | 0.99 | (0.73, 1.31) | 0.95 | (0.72, 1.26) |
|  | | *P value for trend* | |  | *0.265* |  | *0.145* |
| *Potato products (g/day)* | | | |  |  |  |  |
|  | | Q1 (lowest) | |  | Reference |  | Reference |
|  | | Q2 | | 0.92 | (0.68, 1.20) | 0.95 | (0.73, 1.21) |
|  | | Q3 | | 0.81 | (0.61, 1.03) | 0.78 | (0.60, 0.99) |
|  | | Q4 | | 1.02 | (0.77, 1.30) | 0.91 | (0.69, 1.17) |
|  | | Q5 (highest) | | 1.05 | (0.79, 1.37) | 0.95 | (0.71, 1.23) |
|  | | *P value for trend* | |  | *0.204* |  | *0.406* |
| *Roots and tubers (g/day)* | | | |  |  |  |  |
|  | | Q1 (lowest) | |  | Reference |  | Reference |
|  | | Q2 | | 0.97 | (0.73, 1.24) | 1.07 | (0.83, 1.33) |
|  | | Q3 | | 0.78 | (0.60, 1.00) | 0.91 | (0.71, 1.15) |
|  | | Q4 | | 0.77 | (0.58, 0.98) | 1.01 | (0.79, 1.28) |
|  | | Q5 (highest) | | 0.78 | (0.56, 1.02) | 1.05 | (0.80, 1.35) |
|  | | *P value for trend* | |  | *0.006* |  | *0.493* |
| *Pasta (g/day)* | | | |  |  |  |  |
|  | | Q1 (lowest) | |  | Reference |  | Reference |
|  | | Q2 | | 1.00 | (0.77, 1.270 | 1.10 | (0.86, 1.46) |
|  | | Q3 | | 1.05 | (0.81, 1.33) | 1.16 | (0.90,1.49) |
|  | | Q4 | | 0.87 | (0.68, 1.11) | 1.00 | (0.79, 1.26) |
|  | | Q5 (highest) | | 0.82 | (0.64, 1.03) | 1.11 | (0.87, 1.44) |
|  | | *P value for trend* | |  | *0.021* |  | *0.411* |
| *Legumes (g/day)* | | | |  |  |  |  |
|  | Q1 (lowest) | | |  | Reference |  | Reference |
|  | Q2 | | | 0.92 | (0.72, 1.18) | 0.94 | (0.75, 1.18) |
|  | Q3 | | | 0.69 | (0.52, 0.89) | 0.69 | (0.53, 0.87) |
|  | Q4 | | | 1.03 | (0.80, 1.33) | 1.01 | (0.79, 1.28) |
|  | Q5 (highest) | | | 0.93 | (0.72, 1.23) | 0.95 | (0.75, 1.19) |
|  | *P value for trend* | | |  | *0.398* |  | *0.404* |
| *Wholegrains (g/day)* | | | |  |  |  |  |
|  | Q1 (lowest) | | |  | Reference |  | Reference |
|  | Q2 | | | 0.74 | (0.58, 0.93) | 0.83 | (0.66, 1.02) |
|  | Q3 | | | 0.95 | (0.73, 1.22) | 1.03 | (0.80, 1.29) |
|  | Q4 | | | 0.68 | (0.52, 0.89) | 0.78 | (0.59, 1.00) |
|  | Q5 (highest) | | | 0.76 | (0.58, 0.97) | 0.82 | (0.63, 1.04) |
|  | *P value for trend* | | |  | *0.025* |  | *0.080* |

NTTP: Number of teeth with periodontal pocketing>4mm, Q: quintiles; %EI: percent of energy intake.

Mixed-effects negative binomial models were fitted to repeated measurements of the NTPP nested within participants and rate ratios (RR) reported. Model 1 was adjusted for categorical time and continuous energy intake. Model 2 was additionally adjusted for sex, marital status, education, alcohol intake, physical activity, BMI group, self-rated general health, toothbrushing, interdental cleaning and dental attendance.

**Table S9.** The association of baseline starch intake and other macronutrients (kcal/day) with 11-year change in the NTPP among Finnish adults >30 years (2900 observations in 1369 participants).

|  | **Model 1** | | **Model 2** | |
| --- | --- | --- | --- | --- |
|  | **RR** | **(95% CI)** | **RR** | **(95% CI)** |
| Protein (per 25 kcals) | 0.99 | (0.96, 1.02) | 1.00 | (0.97, 1.01) |
| Fat (per 25 kcals) | 1.01 | (1.00, 1.02) | 1.01 | (1.00, 1.01) |
| Sugar (per 25 kcals) | 0.99 | (0.97, 1.00) | 0.99 | (0.98, 1.00) |
| Alcohol (per 25 kcals) | 1.03 | (1.01, 1.06) | 0.99 | (0.97, 1.02) |
| Fibre (per 25 kcals) | 0.95 | (0.83, 1.09) | 1.05 | (0.90, 1.21) |
| Starch (per 25 kcals) | 1.00 | (0.99, 1.02) | 0.99 | (0.98, 1.00) |

NTTP: Number of teeth with periodontal pocketing>4mm, Q: quintiles.

Mixed-effects negative binomial models were fitted to repeated measurements of the NTPP nested within participants. Rate ratios (RR) were reported. Model 1 was adjusted for categorical time and continuous sugar, fibre, proteins, fats and alcohol. Model 2 was additionally adjusted for sex, marital status, education, alcohol intake, physical activity, BMI group, history of hypertension, diabetes, heart disease and stroke, self-rated general health, toothbrushing, interdental cleaning and dental attendance.
